# Exploring the relationship between health literacy and chronic diseases among middle-aged and older adults: evidence from Zhejiang, China

**DOI:** 10.3389/fpubh.2025.1520668

**Published:** 2025-03-25

**Authors:** Shuqi Li, Dingming Yao, Xiujing Hu, Heni Chen, Xiaotong Yan, Yue Xu, Xuehai Zhang

**Affiliations:** ^1^School of Public Health, Hangzhou Normal University, Hangzhou, Zhejiang, China; ^2^Zhejiang Provincial Center for Disease Control and Prevention, Hangzhou, Zhejiang, China

**Keywords:** health literacy, chronic disease, cross-sectional study, middle-aged, older adults, hypertension

## Abstract

**Background:**

Chronic diseases have emerged as a significant public health challenge owing to the escalating global demographic shift toward an aging population. Middle-aged and older individuals are particularly vulnerable to chronic illnesses owing to physiological and socioeconomic changes. By leveraging health literacy data from the Zhejiang Province, this study aimed to elucidate the correlation between health literacy levels and the prevalence of chronic diseases in this demographic cohort.

**Methods:**

In this cross-sectional study, a stratified multistage whole-cluster random sampling method was used to select 12,116 permanent residents aged 45–69 years from 30 monitoring sites in Zhejiang Province from June to November 2023, using the National Health Literacy Monitoring Questionnaire for the Population. Multivariate regression analysis was employed to unravel the correlation between proficiency in health education and the prevention of chronic illnesses.

**Results:**

Sex, age, income, education, self-assessed health status, and smoking status emerged as significant predictors across the different models. Notably, self-assessed health and smoking statuses were identified as confounders that significantly affected the association between health literacy and chronic diseases. Furthermore, this study explored the influence of independent variables on specific chronic diseases, such as hypertension and cerebrovascular disease, with consistent patterns observed across models.

**Conclusion:**

Health literacy is instrumental in thwarting chronic diseases among middle-aged and older individuals. Those with higher levels of health literacy are less likely to suffer from chronic diseases, and high health literacy is a protective factor against hypertension and cerebrovascular disease.

## 1 Introduction

With burgeoning population growth and aging demographics in China, the high incidence of chronic diseases is becoming increasingly obvious, bringing significant challenges and a heavy burden to medical and social security systems. Chronic non-communicable diseases, such as cardiovascular and cerebrovascular diseases, malignant tumors, and diabetes mellitus, have emerged as prominent health threats. According to the World Health Statistics 2023 released by the World Health Organization in May 2023, approximately three-quarters (41 million) of global deaths in 2019 were attributed to chronic diseases (1). Among these, four primary chronic diseases—cardiovascular diseases, cancer, chronic respiratory diseases, and diabetes mellitus— account for approximately 33.3 million deaths. Specifically, there were 17.9 million fatalities owing to cardiovascular diseases, 9.3 million owing to cancer, 4.1 million owing to chronic respiratory diseases, and 2 million owing to diabetes. (2) The proportion of Chinese residents dying of chronic diseases among the total number of deaths was as high as 86.6%, and the associated disease burden reached a critical level. The proportion of older adults is expected to reach 16% by 2030 and 23% by 2050 (3). With the aging of the population and profound lifestyle changes, the incidence of chronic diseases is increasing, showing a trend of rejuvenation, and the prevalence of chronic diseases among middle-aged and older populations is steadily increasing. Chronic diseases have emerged as a significant public health concern, reducing the quality of life of patients and significantly impacting the national economy and social development (4).

Age is a crucial determinant of the manifestation of chronic diseases, with the likelihood of increasing with age. Numerous studies have elucidated a correlation between age and various chronic ailments. For instance, regarding chronic obstructive pulmonary disease, research indicates a surge in hospitalizations with advancing age, particularly among males aged 50 and above (5). Similarly, a Dutch investigation of coronary heart disease highlighted a direct relationship between age and hospital admission for this condition (6). Furthermore, a survey conducted in India examining chronic disease prevalence revealed that individuals in the 45–64 age bracket were most susceptible to developing seven chronic ailments (7). Hence, preventive measures targeting chronic diseases among middle-aged and older individuals can substantially enhance their quality of life, retard disease progression, and promote overall health and well-being.

Health literacy is increasingly crucial in public health. According to the definition given by the U.S. National Library of Medicine, it refers to “an individual’s capacity to access, comprehend, and apply the fundamental health information and services necessary for making informed health decisions.” (8) In the United States, health literacy is considered a long-term objective that is integrated into mid- and long-term health strategies (9). Similarly, European countries articulated health literacy policies early on (10). Health literacy is seen as a key social factor and a public health measure for addressing health inequalities (11). Many countries have incorporated health literacy promotion into various health policies and national strategies, recognizing that health literacy deficiencies may pose significant challenges to individuals and societies (12). In China, the government introduced the policy document “Health Literacy in China: Basic Knowledge and Skills,” which was developed after assessing major health challenges, risk factors, and healthcare needs of the Chinese population. The Chinese government has issued the policy document” Health Literacy for Chinese Citizens: Basic Knowledge and Skills,” which considers the prevalent health issues, risk factors, and healthcare requirements of the population (13). In 2016, China launched the Healthy China 2030 Plan, a national strategic initiative aimed at promoting public health in the medium- and long-term. The importance of health literacy has increased significantly over the past decade. Health literacy is globally acknowledged as the most cost-effective strategy for promoting the well-being of all individuals, and studies have indicated that enhancing health literacy can improve poor lifestyles and behaviors (14, 15).

Current research indicates that approximately 39% of the global population exhibits inadequate health literacy (16), with only 12% of individuals in the United States demonstrating high health literacy levels, 47.6% of individuals in Europe exhibiting insufficient health literacy, and more than half of individuals in Canada lacking adequate health literacy (17–19). The prevalence of inadequate health literacy is widespread. Research has emphasized that persons with restricted health literacy may experience a distorted comprehension of health information, insufficient knowledge of diseases, or lower medication adherence. These factors can contribute to increased mortality risk, diminished health outcomes, and increased healthcare expenditure (20, 21). One study showed a notable increase in health literacy in China, rising from 6.48% in 2008 to 27.78% in 2022, with more than two-thirds of the population classified as having insufficient health literacy by 2022. Health literacy has been demonstrated as a crucial factor in disease management, contributing to favorable health outcomes and enhancing the quality of life for individuals with chronic diseases. Those who are not health literate experience more severe negative disease outcomes than those who are, and individuals with higher levels of health literacy make favorable lifestyle and behavioral choices to improve their health status (22, 23).

Over the recent decades, the conceptualization of health literacy has transcended fundamental reading and numeracy capabilities to incorporate multidimensional competencies essential for effective navigation of the increasingly complex healthcare systems (17). Contemporary frameworks delineate six core dimensions of health literacy: (1) health information processing, (2) foundational medical knowledge, (3) safety protocols and emergency response, (4) chronic disease management, (5) infectious disease prevention, and (6) scientific knowledge encompassing conceptual understanding. Notably, the European Health Literacy Survey has demonstrated cross-cultural validity through standardized implementations in 19 nations as of 2022, with its psychometric properties showing remarkable stability across diverse sociocultural contexts (24). Research has elucidated the pivotal role of health literacy in managing various chronic diseases. For instance, among individuals with hypertension, those with lower health literacy levels exhibited significantly poorer blood pressure control and disease management than their counterparts with higher health literacy (25). Similarly, in individuals diagnosed with diabetes, proficient health literacy is associated with more effective blood glucose control, reduced risk of complications, and improved prognosis (24). Furthermore, individuals with heart failure often lack disease knowledge and demonstrate inadequate adherence to self-care and behavioral practices, leading to exacerbations of their condition or recurrent hospitalizations (26, 27). Hence, integrating health literacy components into chronic disease self-management programs stands out as one of the most efficacious approaches to enhance health outcomes and represents a promising, cost-effective strategy for addressing non-communicable disease challenges.

The current literature predominantly addresses the contemporary landscape of health literacy, focusing on its interplay with demographic and social determinants. Empirical evidence underscores the robust positive correlation between health literacy and health outcomes among older individuals with chronic conditions (28). The presence of one or multiple chronic ailments is concomitant with a heightened comprehension of such conditions, thereby bolstering the overall health literacy levels within the populace (29). Therefore, to address the health threats and economic burdens of chronic diseases, this study examined the relationship between health literacy and chronic diseases among middle-aged and older adults in Zhejiang Province, based on data on health literacy from Zhejiang residents in 2023 and provided a reference for developing and adjusting health promotion strategies.

## 2 Methods

### 2.1 Study design

Initially, the survey was carried out as an integral component of the Health Literacy Monitoring Program in the Zhejiang Province, which is part of the 2023 Chinese Health Literacy Survey. The main aim of this research was to evaluate the health literacy levels of the population. The survey was conducted between June and November 2023. Respondents were non-collective residents of China aged 45 to 69 years, excluding those in military bases, hospitals, prisons, nursing homes, and dormitories. Samples were obtained from 30 monitoring sites in Zhejiang Province using a multistage stratified whole-cluster random sampling method. The permanent resident population was defined as individuals who had resided in the area over 6 months within the last 12 months, regardless of their household registration status. Individuals aged >69 years were excluded because of an increased likelihood of cognitive impairment (30).

The minimum sample size was meticulously calculated for each county, regardless of whether it was a city or a district. The minimum required sample size for each county (city or district) is determined as *N* = $\frac{{\mu_{\alpha }}^{2}*p*\left(1-p\right)}{\delta^{2}}$*deff. Based on the health literacy level of 38.36% of Zhejiang Province residents in 2022 (*p* = 0.3836) and the allowed absolute error (*δ* = 0.3836 × 0.15 = 0.05754), with μα = 1.96 and deff = 1, the final minimum sample size for each county was calculated to be 274. We increased the sample size to 640 per county (city or district) to accommodate invalid questionnaires. A multistage, stratified, probability proportional to size (PPS) sampling method was implemented in adherence to national guidance, consistent with scientific reporting standards (32), and comprised the following steps: (1) Four townships in each county (city or district) were sampled using a stratified, multistage PPS sampling frame; (2) Two neighborhoods (villages) were sampled from each township using the PPS sampling frame; (3) A random sample of 100 households was selected from each neighborhood (village); and (4) one eligible participant from each household was selected for a face-to-face interview using Kish’s grid (32). A minimum of 80 participants from each community (village) completed the questionnaire. Through these sampling steps, 17,545 eligible questionnaires were collected.

Participation in the survey was voluntary, and responses were provided anonymously. Trained interviewers conducted the survey through face-to-face interviews. For strict quality control, data from the questionnaires were subjected to double data entry. The research received approval from the Research Ethics Committee at the Zhejiang Provincial Center for Disease Control and Prevention.

### 2.2 Questionnaire design and health literacy measurement

The assessment of health literacy levels was conducted using the China Health Literacy Scale (31). The China Health Education Center utilized the Delphi method in formulating the scale (13). This comprehensive assessment scale encompassed 50 items categorized across three domains and six sub-dimensions. The three overarching domains are basic knowledge and concepts, healthy lifestyles and behaviors, and health skills. Specifically, the six sub-dimensions are as follows: scientific health concepts, knowledge of infectious diseases, awareness of chronic diseases, proficiency in safety and first aid, understanding of medical treatments, and literacy of health information (33). Sample questions for the various formats of the scale are provided in Appendix Table 1. All questions underwent pretest screening and were categorized into three levels of difficulty were included: easy, medium, and difficult. In the assessment, responses to true-false and single-choice questions were scored either 0 or 1, whereas multiple-choice questions were given a score of either 0 or 2. Overall scores ranged from 0 to 66. Respondents were categorized as possessing sufficient health literacy if they attained a minimum of 80% of the total score, which was equivalent to 53 points (34). Although this convention is commonly embraced in China for ease of interpretation, raw scores were employed for robustness testing purposes. Although this convention is widely adopted in China for ease of interpretation, raw scores were utilized for robustness testing. The scale exhibits internal consistency; the evidence includes a Cronbach’s alpha coefficient of 0.95 and a Spearman-Brown coefficient of 0.94 (35).

### 2.3 Statistical analyses

We collected baseline data related to our participants, including demographic characteristics, lifestyle, dietary habits, and health status. Specifically, we collected key information on age, sex, education level, occupation type, and household income. The health-related component covered the presence of chronic diseases, the type of disease, and the self-assessed health status. To diagnose chronic diseases, we relied on participants’ reports of diagnoses made by a healthcare professional, which included hypertension, heart disease, stroke, diabetes, and cancer. In our analyses, we defined a binary outcome variable, “chronic disease,” to distinguish between participants without a chronic disease and those diagnosed with at least one chronic disease.

Statistical analyses were performed utilizing Stata 18.0. The dependent variables were binary outcomes, and the influence of health literacy was assessed using multiple regression models while accounting for the respondents’ demographics and socioeconomic status indicators. To gain insight into the potential moderating influence of demographic factors, such as gender, age, and income, on the relationship between health literacy and chronic diseases and to assess the degree to which these factors impact outcomes and associations, we performed an interaction analysis. Product terms between health literacy and each moderator (gender, age groups, household income, etc.) were incorporated into multivariable linear regression models. Continuous variables were standardized (*z*-scores), and categorical variables were dummy-coded. The significance of interaction terms was assessed using Wald tests, reporting standardized coefficients (*β*) with 95% confidence intervals. For interpretability, a linear probability model (LPM) was used. To quantitatively assess the impact of various factors on chronic diseases, we constructed a regression model and estimated the parameters using the ordinary least squares (OLS) method. The OLS method is a widely used regression technique that minimizes the sum of squared residuals to determine the relationship between independent variables (predictors) and the dependent variable (outcome). This approach ensures unbiased and efficient estimates under the assumptions of linearity, homoscedasticity, and independence of errors (37). However, in the robustness analysis, we will show that our findings remain consistent when using a nonlinear model, such as the logit model.

## 3 Results

Among the 12,116 participants, men and women accounted for approximately the same proportion. The proportion of middle-aged people aged 40–59 years (58.59%) was greater than that of older people aged 60–69 years (41.41%). A significant number of older individuals suffered from chronic diseases (70.04%). The literacy level of the survey respondents was mostly high school (40.52%), and those suffering from chronic diseases accounted for 35.51%. Those with literacy levels of junior high school and below had a larger proportion of chronic diseases (45.81%). Farmers and other manual laborers were the main patients with chronic diseases, accounting for 83.97% of the patients. The proportion of those with a low family income suffering from chronic diseases was higher (44.20%). Participants with a history of smoking had a higher proportion of chronic diseases, and 78.72% of those who considered themselves healthy had chronic diseases.

The proportion of participants with chronic diseases who were older, had quit smoking, and considered themselves relatively healthy and fit was higher than that of those who did not have chronic diseases. Comparison of differences in the composition of having any chronic disease between demographic groups using the *χ*2 test (Table 1) revealed statistically significant differences between groups in terms of gender, age, education, marital status, occupation, annual household income, smoking status, and self-assessment of health. The *p*-values for all variables were less than 0.01, indicating that these variables were associated with the presence or absence of chronic diseases and could be included as covariates in the subsequent LPM.

**Table 1:** Distribution of participants’ general condition and presence of chronic diseases *n* (%).

Characteristics	All^(p)^	Any chronic diseases ^(s)^		*x* ^2^	*p*
		0	1		
Gender (%)				19.800	<0.001
Male	5,756 (47.51)	3,368 (58.51)	2,388 (41.49)		
Female	6,360 (52.49)	3,973 (62.47)	2,387 (37.53)		
Age (year)				640.987	<0.001
45–59	7,099 (58.59)	4,972 (70.04)	2,127 (29.96)		
60–69	5,017 (41.41)	2,369 (29.96)	2,648 (70.04)		
Education				132.108	<0.001
Less than junior high school	4,704 (38.82)	2,549 (54.19)	2,155 (45.81)		
Junior high school	4,909 (40.52)	3,166 (64.49)	1743 (35.51)		
Senior high school and above	2,503 (20.66)	1,626 (64.96)	877 (35.04)		
Occupation				53.069	<0.001
Personnel of government agencies, enterprises and institutions	847 (6.99)	570 (67.30)	277 (32.70)		
Others	1741 (14.37)	1,002 (57.55)	739 (42.45)		
Farmers	5,993 (49.46)	3,505 (58,48)	2,488 (41.52)		
Factory or manual	2097 (17.31)	1,325 (63.19)	772 (36.81)		
Private enterprises, business (industry) personnel	1,438 (11.87)	939 (65.30)	499 (34.70)		
Household income (Yuan)				54.960	<0.001
<20,000	1,500 (12.38)	837 (55.80)	663 (44.20)		
20,000–79,999	5,478 (45.21)	3,200 (58.42)	2,278 (41.58)		
≥80,000	5,138 (42.41)	3,304 (64.31)	1834 (35.69)		
Region				0.399	0.528
Urban	4,909 (40.52)	2,991 (60.63)	1918 (39.07)		
Rural	7,207 (59.48)	4,350 (60.36)	2,857 (39.64)		
Smoking status				80.969	<0.001
Smoking	2,461 (20.31)	1,509 (61.32)	952 (38.68)		
Have quit smoking	1,337 (10.03)	659 (49.29)	678 (50.71)		
Not smoking	8,318 (68.66)	5,173 (62.19)	3,145 (37.81)		
Self-assessed health status				859.370	<0.001
Poor	3,596 (29.68)	2,717 (75.56)	879 (24.44)		
Relatively poor	3,737 (30.84)	2,379 (63.66)	1,358 (36.34)		
Fair	4,123 (34.04)	2069 (50.18)	2054 (49.82)		
Relatively good	519 (4.28)	146 (28.13)	373 (71.87)		
Good	141 (1.16)	30 (21.28)	111 (78.72)		

(p) Percentage; (s), 1 if there is any chronic disease, 0 otherwise.

Statistical analyses revealed a consistently negative and significant main effect of health literacy on the risk of chronic diseases across all models. Interaction analyses revealed that demographic characteristics had significant moderating effects. First, gender significantly moderated the protective effect of health literacy (interaction term *β* = −0.063, *p* = 0.004), with stratified analyses showing stronger negative associations in males than in females. Second, age group exhibited differential moderating effects, with attenuated protective effects observed in the 60–69-years subgroup (interaction term *β* = 0.052, *p* = 0.029). Household income had a significant moderating effect in only the high-income group (*β* = 0.041, *p* = 0.038). Notably, no significant interaction effects were observed between health literacy and educational attainment, occupational categories, or self-rated health status (all *p* > 0.05). However, smoking status revealed a distinct moderation pattern, with significantly enhanced negative effects of health literacy in non-smokers (interaction term *β* = −0.071, *p* = 0.012) (Table 2).

**Table 2:** Analysis of the interaction effect of health literacy with demographic variables.

Variable	Coefficient (SE)	*t*-value	*p*-value	95% CI
Health Literacy (Adequate vs. inadequate)	−0.058*** (0.015)	−3.74	<0.001	[−0.088, −0.027]
F01 (Group male vs. female)	−0.028** (0.010)	−2.80	0.005	[−0.047, −0.008]
Health Literacy × F01	−0.063** (0.022)	−2.89	0.004	[−0.105, −0.020]
Health Literacy (Adequate vs. inadequate)	−0.055*** (0.013)	−4.34	<0.001	[−0.080, −0.030]
Age group (Aged 60–69 vs. Aged 45–59 years)	0.213*** (0.010)	21.87	<0.001	[0.194, 0.233]
Health Literacy × Age group	0.052* (0.024)	2.19	0.029	[0.005, 0.099]
Health Literacy (Adequate vs. inadequate)	−0.147*** (0.038)	−3.86	<0.001	[−0.221, −0.072]
Household income (20000–79,999 yuan vs. <20,000 yuan)	–0.032* (–0.015)	−2.12	0.034	[−0.062, −0.002]
Household income (>80,000 yuan vs. <20,000 yuan)	−0.084*** (0.016)	−5.37	<0.001	[−0.115, −0.053]
Health Literacy × Household income (20000–79,999 yuan)	0.069 (0.042)	1.63	0.102	[−0.014, 0.151]
Health Literacy × Household income (>80,000 yuan)	0.081* (0.040)	1.99	0.046	[0.001, 0.161]
Health Literacy (adequate vs. inadequate)	−0.083* (0.034)	−2.48	0.013	[−0.149, −0.017]
Occupation (Others vs. Personnel of government agencies, enterprises and institutions)	0.074** (0.028)	2.69	0.007	[0.020, 0.128]
Occupation (Farmers vs. Personnel of government agencies, enterprises and institutions)	0.052* (0.025)	2.04	0.041	[0.002, 0.128]
Occupation (Factory or manual vs. Personnel of government agencies, enterprises and institutions)	0.021 (0.027)	0.77	0.440	[−0.032, 0.074]
Occupation (Private enterprises, business [industry] personnel vs. Personnel of government agencies, enterprises and institutions)	−0.006 (0.029)	−0.19	0.846	[−0.063, 0.051]
Health Literacy × Occupation (Others)	−0.021 (0.044)	−0.48	0.635	[−0.108, 0.066]
Health Literacy × Occupation (Farmers)	0.028 (0.038)	0.73	0.467	[−0.047, 0.102]
Health Literacy × Occupation (Factory or manual)	−0.024 (0.042)	−0.58	0.562	[−0.107, 0.058]
Health Literacy × Occupation (Private enterprises, business [industry] personnel)	0.030 (0.043)	0.70	0.481	[−0.054, 0.115]
Health Literacy (Adequate vs. inadequate)	−0.065* (0.027)	−2.39	0.017	[−0.118, −0.012]
Education (Junior high school vs. less than junior high school)	−0.101*** (0.011)	−9.40	<0.001	[−0.122, −0.080]
Education (Senior high school and above vs. less than junior high school)	−0.066*** (0.015)	−4.37	<0.001	[−0.096, −0.037]
Health Literacy × Education (Junior high school)	0.032 (0.032)	1.01	0.313	[−0.030, 0.946]
Health Literacy × Education (Senior high school and above)	−0.034 (0.033)	−1.03	0.304	[−0.099, 0.031]
Health Literacy (Adequate vs. inadequate)	−0.068*** (0.019)	−3.57	<0.001	[−0.105, −0.031]
Self-assessed health (Relatively poor vs. poor)	0.127*** (0.013)	10.15	<0.001	[0.103, 0.152]
Self-assessed health (Fair vs. poor)	0.250*** (0.012)	20.79	<0.001	[0.226, 0.274]
Self-assessed health (Relatively good vs. poor)	0.459*** (0.024)	19.26	<0.001	[0.413, 0.506]
Self-assessed health (Good vs. poor)	0.518*** (0.042)	12.27	<0.001	[0.435, 0.600]
Health Literacy × Self-assessed health (Relatively poor)	−0.025 (0.026)	−0.94	0.345	[−0.076, 0.026]
Health Literacy × Self-assessed health (Fair)	0.010 (0.027)	0.37	0.711	[−0.042, 0.062]
Health Literacy × Self-assessed health (Relatively good)	0.070 (0.064)	1.09	0.274	[−0.056, 0.196]
Health Literacy × Self-assessed health (Good)	0.200 (0.149)	1.34	0.179	[−0.092, 0.492]
Health Literacy (Adequate vs. inadequate)	−0.041 (0.025)	−1.62	0.106	[−0.090, 0.009]
Smoking status (Have quit smoking vs. smoking)	0.118*** (0.019)	6.37	<0.001	[0.082, 0.154]
Smoking status (Not smoking vs. smoking)	0.008 (0.012)	0.65	0.515	[−0.016, 0.032]
Health Literacy × Smoking status (Have quit smoking)	0.016 (0.041)	0.40	0.692	[−0.064, 0.096]
Health Literacy × Smoking status (Not smoking)	−0.071* (0.028)	−2.50	0.012	[−0.126, −0.015]

*** *p* < 0.001, ** *p* < 0.01, * *p* < 0.05. CI, confidence interval.

We predicted the occurrence of chronic diseases using the hierarchical equations outlined in Table 3. In Model 1, we excluded any covariates and solely incorporated the binary variable “health literacy.” For Models 2–4, we progressively incorporated three sets of variables into the equation: sex, age group, and household income; occupation, education; and self-assessed health status and smoking status. This provided an opportunity to observe how each block is presented in Table 3. The second equation in Model 2, which incorporates sex, age group, and annual income, revealed that sex is associated with the likelihood of chronic diseases. Specifically, females exhibit a higher propensity to develop chronic diseases than males. The effects of age and household income were also significant, showing that older and high-income respondents were likely to report having chronic diseases. The negative effect of health literacy persisted, albeit with a slight decrease in its magnitude.

**Table 3:** OLS estimates on having any chronic disease.

Dep: Has any chronic disease	Model 1	Model 2	Model 3	Model 4	Model 5
Sample	All	All	All	All	All
Adequate health literacy (=1)	−8.17*** (<0.001)	−3.50*** (<0.001)	−5.01*** (<0.001)	−6.69*** (<0.001)	−2.32** (0.020)
Gender (=1 female)		−4.92*** (<0.001)			−4.71*** (<0.001)
Age group (Base: Aged 45–59)					
Aged 60–69		24.23*** (<0.001)			20.60*** (<0.001)
Household income (Base: <20000 yuan)					
20,000-79999 yuan		−0.68 (0.494)			1.59 (0.112)
≥80000 yuan		−1.85* (0.064)			1.62 (0.106)
Occupation (Based: Personnel of government agencies, enterprises and institutions)					
Others			2.73*** (0.006)		1.46 (0.144)
Farmers			1.95* (0.051)		−0.57 (0.571)
Factory or manual			0.47 (0.638)		−0.38 (0.702)
Private enterprises, business (industry) personnel			0.45 (0.654)		0.17 (0.862)
Education (Based: Less than junior high school)					
Junior high school			−8.92*** (<0.001)		−3.69*** (<0.001)
Senior high school and above			−4.58*** (<0.001)		−2.52** (0.012)
Self-assessed health status (Based: Poor)					
Relatively poor				10.95*** (<0.001)	9.94*** (<0.001)
Fair				23.39*** (<0.001)	22.12*** (<0.001)
Relatively good				21.19*** (<0.001)	19.76*** (<0.001)
Good				12.93*** (<0.001)	12.10*** (<0.001)
Smoking status (Based: Smoking)					
Have quit smoking				5.97*** (<0.001)	4.80*** (<0.001)
Not smoking				−2.14** (0.032)	0.77 (0.440)
Observations	12,116	12,116	12,116	12,116	12,116
R-squared	0.0055	0.0562	0.0146	0.0800	0.1186

The dependent variable is a binary variable indicating whether a respondent has any chronic disease (=1 if there is any chronic disease and 0 otherwise). OLS, ordinary least squares. Model 1: includes adequate health literacy. Model 2: gender, age group, and household income in addition to the variable in Model 1. Model 3: occupation and education in addition to the variable in Model 1. Model 4: self-assessed health status and smoking status in addition to the variable in Model 1. Model 5: variables in Models 1–4. Estimates of the constants have not been reported. *** *p* < 0.01, ** *p* < 0.05, * *p* < 0.1. Standard errors are shown in parentheses.

Model 3 incorporates occupation and education. The findings from Model 3 revealed that education also emerged as a significant determinant of a respondent’s chronic condition. These findings imply that increased educational attainment correlates with a reduced probability of chronic illnesses. Compared with personnel of government agencies, enterprises, and institutions, farmers and others have a higher probability of developing chronic diseases. The statistical and economic significance of these effects suggests their importance as predictors of chronic diseases.

In Model 4, we included self-assessed health and smoking status. The results indicated that individuals who perceived themselves as being in better health were paradoxically more prone to chronic disease, and those who had quit smoking were more likely to have chronic diseases than current smokers.

Furthermore, a significant increase in the R-squared value was observed in Model 4 compared to Models 1 through 3. This suggests that self-assessed health status and smoking status are primary confounding variables that influence the relationship between health literacy and chronic diseases, as observed in Model 1. Model 5 included the full set of covariates. We found that the effect of household income and occupation on chronic diseases disappeared, and the R-squared increased further.

We applied a consistent methodology to investigate the influence of independent variables, such as health literacy, on various types of chronic diseases. The findings from our hypertension study closely paralleled those detailed in Table 3. Specifically, Model 2 illustrated a heightened propensity for hypertension among men and the older population. Model 3 elucidated an increased likelihood of hypertension among individuals engaged in farming or other occupations, as well as among those with low literacy levels. Moreover, Model 4 highlights a greater probability of hypertension among individuals who perceive themselves to be in better health and former smokers (Table 4).

**Table 4:** OLS estimates on having hypertension.

Dep: Has hypertension	Model 1	Model 2	Model 3	Model 4	Model 5
Sample	All	All	All	All	All
Adequate health literacy (=1)	−7.45*** (<0.001)	−3.30*** (0.001)	−4.38*** (<0.001)	−6.69*** (<0.001)	−2.00** (0.045)
Gender(=1 female)		−4.84*** (<0.001)			−4.16*** (<0.001)
Age group (Base: Aged 45–59)					
Aged 60–69		21.86*** (<0.001)			18.79*** (<0.001)
Household income (Base: <20000 yuan)					
20,000–79999 yuan		−0.85 (0.397)			1.96* (0.050)
≥80000 yuan		−0.35 (0.726)			1.70* (0.089)
Occupation (Based: Personnel of government agencies, enterprises and institutions)					
Others			2.60*** (0.009)		1.49 (0.136)
Farmers			2.07** (0.039)		0.14 (0.891)
Factory or manual			1.13 (0.258)		0.42 (0.677)
Private enterprises, business (industry) personnel			0.58 (0.561)		0.34 (0.730)
Education (Based:1: Less than junior high school)					
Junior high school			−8.64*** (<0.001)		−4.44*** (<0.001)
Senior high school and above			−4.46*** (<0.001)		−2.94*** (0.003)
Self-assessed health status (Based: Poor)					
Relatively poor				7.22*** (<0.001)	6.25*** (<0.001)
Fair				15.91*** (<0.001)	14.53*** (<0.001)
Relatively good				10.66*** (<0.001)	9.14*** (<0.001)
Good				7.04*** (<0.001)	6.09*** (<0.001)
Smoking status (Based: Smoking)					
Have quit smoking				4.88*** (<0.001)	3.79*** (<0.001)
Not smoking				−1.96* (0.050)	0.55 (0.580)
Observations	12,116	12,116	12,116	12,116	12,116
R-squared	0.0046	0.0462	0.0128	0.0351	0.0706

The dependent variable is a binary variable indicating whether a respondent has hypertension (=1 if hypertensive; otherwise, 0). OLS, ordinary least squares. Model 1: includes adequate health literacy. Model 2: gender, age group, and household income in addition to the variable in Model 1. Model 3: occupation and education in addition to the variable in Model 1. Model 4: self-assessed health status and smoking status in addition to the variable in Model 1. Model 5: variables in Models 1–4. Estimates of the constants have not been reported. *** *p* < 0.01, ** *p* < 0.05, * *p* < 0.1. Standard errors are shown in parentheses.

Additionally, our investigation revealed a notable impact of occupation on the development of hypertension, further emphasizing its significance. Models 1 and 5 exhibited significant and consistent trends for cerebrovascular disease. This suggests that high literacy continues to serve as a protective factor for cerebrovascular disease, even after controlling for all confounding variables, as indicated by the findings (Table 5). The same methodology did not reveal any interaction between independent variables, such as health literacy and diabetes (Table 6).

**Table 5:** OLS estimates on having cerebrovascular disease.

Dep: Has cerebrovascular disease	Model 1	Model 2	Model 3	Model 4	Model 5
Sample	All	All	All	All	All
Adequate health literacy (=1)	−2.65*** (0.008)	−0.21 (0.832)	−1.07 (0.285)	−1.46 (0.144)	−2.46** (0.014)
Gender (=1 female)		−0.42 (0.675)			−2.88*** (0.004)
Age group (Base: Aged 45–59)					
Aged 60–69		10.35*** (<0.001)			3.58*** (<0.001)
Household income (Base: <20000 yuan)					
20,000–79999 yuan		−1.73* (0.083)			0.25 (0.799)
≥80000 yuan		−3.00*** (0.003)			−0.78 (0.435)
Occupation (Based: Personnel of government agencies, enterprises and institutions)					
Others			0.19 (0.852)		0.04 (0.969)
Farmers			1.28 (0.202)		−0.43 (0.670)
Factory or manual			−0.18 (0.860)		−1.13 (0.260)
Private enterprises, business (industry) personnel			0.49 (0.622)		0.01 (0.993)
Education (Based:1: Less than junior high school)					
Junior high school			−3.96*** (<0.001)		0.92 (0.360)
Senior high school and above			−2.40** (0.016)		0.16 (0.871)
Self-assessed health status (Based: Poor)					
Relatively poor				3.20*** (0.001)	1.49 (0.135)
Fair				9.84*** (<0.001)	6.42*** (<0.001)
Relatively good				15.16*** (<0.001)	12.04*** (<0.001)
Good				10.69*** (<0.001)	10.22*** (<0.001)
Smoking status (Based: Smoking)					
Have quit smoking				2.88*** (0.004)	2.59** (0.010)
Not smoking				−1.98** (0.048)	−0.34 (0.736)
Observations	12,116	12,116	12,116	12,116	12,116
R-squared	0.0006	0.0113	0.0029	0.0315	0.0284

The dependent variable is a binary variable indicating whether a respondent has cerebrovascular disease (=1 if there is cerebrovascular disease and 0 otherwise). OLS, ordinary least squares. Model 1: includes adequate health literacy. Model 2: gender, age group, and household income in addition to the variable in Model 1. Model 3: occupation and education in addition to the variable in Model 1. Model 4: self-assessed health status and smoking status in addition to the variable in Model 1. Model 5: variables in Models 1–4. Estimates of the constants have not been reported. *** *p* < 0.01, ** *p* < 0.05, * *p* < 0.1. Standard errors are shown in parentheses.

**Table 6:** OLS estimates on having diabetes.

Dep: Has diabetes	Model 1	Model 2	Model 3	Model 4	Model 5
Sample	All	All	All	All	All
Adequate health literacy (=1)	−3.93*** (<0.001)	−1.16 (0.247)	−2.14** (0.032)	−2.84*** (0.005)	−0.38 (0.702)
Gender(=1 female)		−3.51*** (<0.001)			−2.81*** (0.005)
Age group (Base: Aged 45–59)					
Aged 60–69		14.09*** (<0.001)			11.68*** (<0.001)
Household income (Base: <20000 yuan)					
20,000–79999 yuan		−1.82* (0.069)			−0.30 (0.766)
≥80000 yuan		−2.07** (0.038)			−0.12 (0.905)
Occupation (Based: Personnel of government agencies, enterprises and institutions)					
Others			0.51 (0.608)		−0.20 (0.844)
Farmers			−0.12 (0.907)		−1.59 (0.113)
Factory or manual			−1.31 (0.191)		−1.78* (0.075)
Private enterprises, business (industry) personnel			−0.69 (0.488)		−0.83 (0.404)
Education (Based:1: Less than junior high school)					
Junior high school			−5.71*** (<0.001)		–2.57** (0.010)
Senior high school and above			−3.48*** (0.001)		–2.28** (0.023)
Self-assessed health status (Based: Poor)					
Relatively poor				6.35*** (<0.001)	5.68*** (<0.001)
Fair				12.84*** (<0.001)	11.92*** (<0.001)
Relatively good				13.17*** (<0.001)	12.08*** (<0.001)
Good				8.69*** (<0.001)	8.04*** (<0.001)
Smoking status (Based: Smoking)					
Have quit smoking				1.66* (0.097)	0.90 (0.366)
Not smoking				−2.94*** (0.003)	−0.86 (0.392)
Observations	12,116	12,116	12,116	12,116	12,116
R-squared	0.0013	0.0197	0.0051	0.0285	0.0427

The dependent variable is a binary variable indicating whether the respondent has diabetes (=1 if has diabetes and 0 otherwise). OLS, ordinary least squares. Model 1: includes adequate health literacy. Model 2: gender, age group, and household income in addition to the variable in Model 1. Model 3: occupation and education in addition to the variable in Model 1. Model 4: self-assessed health status and smoking status in addition to the variable in Model 1. Model 5: variables in Models 1–4. Estimates of the constants have not been reported. *** *p* < 0.01, ** *p* < 0.05, * *p* < 0.1. Standard errors are shown in parentheses.

## 4 Discussion

The primary objective of this investigation was to examine the correlation between health literacy levels and the prevalence of chronic diseases in the middle-aged and older population of China and to analyze the influence of demographic attributes on this relationship. Our findings indicate that, as of 2023, the chronic disease prevention literacy rate among middle-aged and older residents of Zhejiang Province was 26.44%, with a health literacy rate of 21.20% (Table 7). This implies that approximately 75% of individuals aged 45–69 lack the ability to access, comprehend, and utilize health-related information and services and independently make informed health decisions (18). There remains a considerable disparity in health literacy levels between middle-aged and older individuals compared to those aged 15–69.

**Table 7:** Health literacy of six categories of participants.

Categories	*n*	%
Health information literacy	3,946	32.57
Basic medical literacy	2,633	21.73
Safety and first aid literacy	6,405	52.80
Chronic disease prevention literacy	3,203	26.44
Infectious disease prevention literacy	2,366	19.53
Scientific view of health	5,817	48.01

Chronic diseases represent the foremost threat to human life and well-being, with an escalating incidence in recent years, particularly among middle-aged and older patients. The onset of chronic illnesses frequently precipitates physical and psychological ailments, functional impairment, and accelerated aging, often compounded by improper medication usage, and other factors, leading to the manifestation of multiple chronic conditions (62). Extensive research underscores the heightened risk of adverse health outcomes, including mortality, associated with the co-occurrence of multiple chronic ailments (36, 38).

Consistent with previous studies, our analysis revealed a significant positive correlation between health literacy and health outcomes among older individuals with chronic illnesses (23, 62). Health literacy concerns an individual’s capacity to access, comprehend, and apply health-related information, exerts a profound influence on the etiology and trajectory of chronic illnesses (39). It has been proposed that health literacy, as a competency in accessing health-related information, may directly or indirectly impact the capability of middle-aged and older adults to access preventive measures for chronic diseases, aligning with scientific discourse. Consequently, enhancing health literacy levels in middle-aged and older cohorts has emerged as a pivotal strategy for mitigating the health ramifications of chronic diseases. Elevating health literacy levels engender a proclivity toward adopting healthier lifestyles and behaviors, thereby forestalling the onset and progression of chronic diseases. Inadequate health literacy is also a major barrier to health education for people with chronic diseases, making it difficult to improve their lifestyle and participate in treatment decisions (40). Health literacy has emerged as a more potent predictor of population health status than socioeconomic status, age, and ethnicity (41).

Age, education, marital status, monthly income, and the number of diseases were key determinants in surveys of chronic disease prevalence and health literacy among middle-aged and older adults in various Chinese provinces, showing clear regional differences. In the 2018–2019 survey in Jilin Province, education played a key role in the manifestation of chronic diseases in the target population, whereas the impact of household income was only evident in 2019 (42). A 2019 study in Shanghai in 2019 highlighted the significant influence of educational attainment and self-assessed health status on health literacy in middle-aged and older populations (43). A 2019 survey of the middle-aged and older Kazakh population in the Ili region of Xinjiang showed that household classification, educational attainment, average monthly income, prevalence of chronic diseases, and self-reported health status influenced health literacy in this population (40). In 2022, a study of health literacy levels of residents aged 15–69 years in Zhejiang Province, considering various chronic disease prevalences, highlighted the number of chronic disease types as a key factor influencing residents’ health literacy levels. It also noted that patients with multiple chronic diseases often face a higher risk of inadequate health literacy (44).

Demographic characteristics play a crucial role in the association between health literacy and chronic diseases. Consistent with the findings of other studies, there was a negative correlation between women and chronic diseases, suggesting a lower prevalence in women (45). This trend may stem from women’s greater propensity to seek health knowledge and monitor their health more than men. In addition, the negative association between health literacy and chronic diseases among middle-aged and older adults implies a higher likelihood of chronic diseases among those with lower health literacy in these age groups. Previous research emphasized that older individuals, particularly those with lower educational attainment and health literacy, are more susceptible to chronic diseases. This vulnerability may arise from factors such as decreased receptiveness to knowledge and information, limited engagement with external sources, and fewer avenues for accessing health-related information.

Chronic diseases in older adults typically have lifelong, protracted courses and poor prognoses. Low health literacy negatively affects disease prevention, self-management, and clinical treatment, thereby influencing health outcomes through improvements in health behaviors. In contrast, middle-aged individuals are in active life and career development stages, with greater access to diverse health information channels such as the Internet, health apps, and social media. They are adept at translating this information into changes in health behaviors and supporting health decision-making. However, with aging, individuals face physical and cognitive challenges such as reduced reading ability and memory loss, hindering their access to and comprehension of healthcare information. Given that chronic diseases in older adults often require long-term treatment and management, a high level of health literacy is essential for effective coping.

Consistent with the existing research, our study underscores the strong relationship between socioeconomic factors, including education level, and chronic diseases. This highlights the importance of considering socioeconomic factors when formulating health policies. We found that the prevalence of chronic diseases was significantly higher in the low-income group than in other income strata. This disparity may be attributed to poor lifestyle habits and limited access to healthcare services. Furthermore, persistent economic stress and the absence of social support negatively affect health status.

Conversely, having a stable economic base allows access to high-quality healthcare services and promotes proactive health management. Patients with low literacy are less capable of reading, analyzing problems, and filtering information and cannot cooperate effectively with doctors for treatment and disease self-management. Those with higher educational levels often feel supported by healthcare providers and have access to adequate health information. This may be attributed to their enhanced confidence in communicating with healthcare providers, which leads to greater feelings of understanding and support (46). Efforts to improve health literacy levels among middle-aged and older populations should involve targeted interventions such as health education, family support, and community care. Additionally, initiatives aimed at enhancing education, improving occupational environments, and strengthening social security systems can effectively reduce the risk of disease in these populations. Enhancing health literacy can improve health outcomes for patients with chronic diseases (47).

According to a 2023 World Health Organization report, tobacco consumption is closely linked to chronic diseases (48). Middle-aged and older individuals without a smoking history show heightened concerns about self-care, physical well-being, and mental health (49). They are proactive in utilizing healthcare services, accessing medical support and information, and effectively managing their conditions. Studies have indicated that individuals without chronic diseases often report higher self-assessed health scores, which is consistent with our findings. These scores serve as composite indicators of physical and mental health status and independently predict future health outcomes, including mortality (50, 51). Health literacy research across various countries, such as the United States, Europe, the Netherlands, Canada, Japan, and China, consistently links higher health literacy with better self-rated health among older adults (17, 52–55).

Nevertheless, a dearth of comprehensive exploration regarding the correlation between self-rated health and chronic diseases remains. Self-rated health has been established as an independent predictor of mortality and overall health outcomes (50). It has also been proven to be a potent predictor of mortality in individuals with diagnosed chronic diseases, but not a predictor of chronic disease incidence (56). Furthermore, patients with chronic obstructive pulmonary disease are reported to exhibit poorer self-rated health compared to healthy controls (57). We conducted an in-depth study to reveal the association between health literacy levels and the prevalence of various chronic diseases. Notably, middle-aged and older adults with higher health literacy, particularly those with hypertension and cerebrovascular disease, showed a reduced likelihood of developing these conditions. This association remained significant even after adjusting for potential confounding variables. These findings underscore the crucial role of health literacy in chronic disease prevention and management, highlighting the need for future health policies and interventions to improve among middle-aged and older populations (49). Hypertension is a major chronic disease that significantly affects population health and health outcomes. Studies focusing on patients with hypertension have demonstrated that those with higher health literacy exhibit better hypertension control and are more responsive to externally provided effective health education and lifestyle guidance. Moreover, studies have linked higher HL to a decreased risk of ischemic cardiovascular disease and enhanced health-related quality of life among individuals with hypertension.

Health literacy, conceptualized through the tripartite framework of knowledge-belief-action theory, operates through interconnected cognitive and behavioral dimensions where knowledge acquisition serves as the foundational element, attitudinal transformation acts as the motivational catalyst, and health-promoting behaviors constitute the ultimate objective. This hierarchical progression suggests a dose–response relationship, that is, cumulative health knowledge crystallizes into health beliefs, which subsequently drive behavioral modifications (58). Enhancing health education and patient empowerment is a crucial strategy for the prevention and management of hypertension, with health literacy playing a pivotal role. Our study revealed that educational attainment exhibited a negative association with the onset of hypertension. Previous research has shown that individuals with limited health literacy often lack sufficient knowledge of preventive healthcare, struggle with self-management skills, and face challenges in comprehending medical advice and adhering to medication regimens. These difficulties ultimately affect their health status, disease prognosis, and healthcare expenditures. Given the heightened health awareness among hypertensive patients coupled with their proactive approach to seeking health knowledge and accessing health education services during medical encounters, there is an opportunity for dynamic improvements in health literacy. Personal experiences in healthcare settings and engagement with medical and health services significantly contribute to literacy levels. In summary, prioritizing health education and improving health literacy are vital strategies in a comprehensive approach to hypertension prevention and control, ultimately resulting in improved health outcomes and decreased healthcare expenditures.

According to the health literacy-health outcomes causal model, health literacy should be viewed as both a patient and system phenomenon. First, individuals with adequate health literacy are more likely to interpret nutritional labels, understand public health guidelines, and adopt preventive measures, as evidenced in longitudinal studies linking health literacy to reduced obesity and hypertension rates (59). Second, enhanced disease awareness enables early risk identification; for example, health-literate populations demonstrate greater familiarity with disease biomarkers, such glycated hemoglobin for diabetes or blood pressure thresholds, prompting timely screening (60). Third, better health management skills, including medication adherence, symptom monitoring, and patient-provider communication, empower individuals to navigate the complex healthcare systems (61).

These findings hold significant implications for public health policies and practices. Given the evident link between the health literacy levels of middle-aged and older populations and the prevalence of chronic diseases, it is imperative for the government and society to prioritize the enhancement of health literacy among these demographics. This can be achieved through robust health education initiatives to foster healthy lifestyles and disseminate accurate health information. Through these means, we can effectively prevent and control chronic diseases, thereby easing the strain on healthcare systems. This focus should especially target individuals with lower levels of education, those engaged in occupations such as farming or manual labor, and those with limited household income. Targeted health education programs and services tailored to the specific needs of these groups can significantly reduce their disease susceptibility.

It is crucial to recognize the limitations of this study. First, its reliance on a cross-sectional survey design precludes causality. Second, although previous research highlighted regional disparities in health literacy levels across China, focusing solely on the middle-aged and older populations in Zhejiang Province limits the generalizability of our findings. Future research should employ a longitudinal design to ascertain the causal relationship between health literacy and chronic diseases. Additionally, exploring the efficacy of different interventions for health literacy and chronic diseases will further enrich our understanding of this field.

## 5 Conclusion

Our study underscores the pivotal role of health literacy in preventing chronic diseases among middle-aged and older adults. Individuals with higher levels of health literacy exhibited a decreased likelihood of experiencing chronic diseases, underscoring the significance of bolstering health education and literacy initiatives. Our analysis accentuates the multifaceted nature of chronic diseases, wherein demographic, socioeconomic, and behavioral factors synergistically influence their onset. Furthermore, our findings indicate that enhanced health literacy serves as a protective shield against hypertension and cerebrovascular disease. These insights provide an important reference for healthcare workers, enabling physicians to design personalized treatment plans better and preventive measures, improve patients’ health literacy, reduce the prevalence of chronic diseases, and improve treatment outcomes; it also provides an important basis for public health policymakers.

## Data Availability

The original contributions presented in the study are included in the article/Supplementary material, further inquiries can be directed to the corresponding authors.
