# Supplementary material for: Exploring the relationship between health literacy and chronic diseases among middle-aged and older adults: evidence from Zhejiang, China
Source: Front Public Health. 2025 Mar 25;13:1520668. doi: 10.3389/fpubh.2025.1520668 (PMC11975670; doi:10.3389/fpubh.2025.1520668)
Supplement: Supplementary file 1 [file Table_1.docx]

**Supplementary Table 1**  OLS estimates on having cardiac disease

| Dep: Has Cardiac disease | Model 1 | Model 2 | Model 3 | Model 4 | Model 5 |
| --- | --- | --- | --- | --- | --- |
| Sample | All | All | All | All | All |
| Adequate health literacy (=1) | –2.65***  (0.008) | -0.21  (0.832) | –1.07  (0.285) | –1.46  (0.144) | 0.24  (0.807) |
| Gender (=1 female) |  | 0.42  (0.675) |  |  | –1.65*  (0.099) |
| Age group (Base: Aged 45–59) |  |  |  |  |  |
| Aged 60-69 |  | 10.35***  (<0.001) |  |  | 8.45***  (<0.001) |
| Household income (Base: <20000 yuan) |  |  |  |  |  |
| 20000-79999 yuan |  | –1.73*  (0.083) |  |  | 0.04  (0.967) |
| ≥80000 yuan |  | –3.00***  (0.003) |  |  | -0.44  (0.663) |
| Occupation (Base: Personnel of government agencies, enterprises and institutions) |  |  |  |  |  |
| Others |  |  | 0.19  (0.852) |  | -0.46  (0.645) |
| Farmers |  |  | 1.28  (0.202) |  | –0.15  (0.880) |
| Factory or manual |  |  | -0.18  (0.860) |  | –0.49  (0.627) |
| Private enterprises, business (industry) personnel |  |  | 0.49  (0.622) |  | 0.47  (0.639) |
| Education (Base: Less than junior high school) |  |  |  |  |  |
| junior high school |  |  | –3.96***  (0.001) |  | –0.68  (0.497) |
| senior high school and above |  |  | –2.40**  (0.016) |  | –0.61  (0.539) |
| Self-assessed health status (Base: Poor) |  |  |  |  |  |
| Relatively poor |  |  |  | 3.20***  (0.001) | 2.66***  (0.008) |
| Fair |  |  |  | 9.84***  (<0.001) | 9.07***  (<0.001) |
| Relatively good |  |  |  | 15.16***  (<0.001) | 14.20***  (<0.001) |
| Good |  |  |  | 10.69***  (<0.001) | 10.18***  (<0.001) |
| Smoking status (Base: Smoking) |  |  |  |  |  |
| Have quit smoking |  |  |  | 2.88***  (0.004) | 2.37**  (0.018) |
| Not smoking |  |  |  | 1.98**  (0.048) | 2.41**  (0.016) |
| Observations | 12116 | 12116 | 12116 | 12116 | 12116 |
| R-squared | 0.0006 | 0.0113 | 0.0029 | 0.0315 | 0.0384 |

Note: The dependent variable is a binary variable indicating whether the respondent has Cardiac disease (=1 if has Cardiac disease and 0 otherwise). OLS: ordinary least squares. Model 1 includes adequate health literacy. Model 2: gender, age group, and household income in addition to the variable in Model 1. Model 3: occupation and education in addition to the variable in Model 1. Model 4: self-assessed health status and smoking status in addition to the variable in Model 1. Model 5: variables in Models 1–4. Estimates of the constants have not been reported. ***p < 0.01, ** p < 0.05, * p < 0.1. Standard errors are shown in parentheses.
