# Supplementary material for: Exploring the relationship between health literacy and chronic diseases among middle-aged and older adults: evidence from Zhejiang, China
Source: Front Public Health. 2025 Mar 25;13:1520668. doi: 10.3389/fpubh.2025.1520668 (PMC11975670; doi:10.3389/fpubh.2025.1520668)
Supplement: Supplementary file 2 [file Table_2.docx]

**Supplementary Table 2**  OLS estimates on having malignant tumor

| Dep: Has Malignant tumor | Model 1 | Model 2 | Model 3 | Model 4 | Model 5 |
| --- | --- | --- | --- | --- | --- |
| Sample | All | All | All | All | All |
| Adequate health literacy (=1) | 1.18  (0.237) | 1.72*  (0.086) | 1.31  (0.191) | 2.09**  (0.037) | 1.56  (0.120) |
| Gender (=1 female) |  | 3.18***  (0.001) |  |  | 2.40**  (0.016) |
| Age group (Base: Aged 45–59) |  |  |  |  |  |
| Aged 60-69 |  | 1.50  (0.132) |  |  | 0.37  (0.713) |
| Household income (Base: <20000 yuan) |  |  |  |  |  |
| 20000-79999 yuan |  | –1.86*  (0.062) |  |  | -0.47  (0.641) |
| ≥80000 yuan |  | –2.08**  (0.038) |  |  | -0.26  (0.793) |
| Occupation (Base: Personnel of government agencies, enterprises and institutions) |  |  |  |  |  |
| Others |  |  | -0.92  (0.356) |  | -1.15  (0.248) |
| Farmers |  |  | 10.01  (0.993) |  | –0.52  (0.603) |
| Factory or manual |  |  | -1.03  (0.304) |  | –1.02  (0.310) |
| Private enterprises, business (industry) personnel |  |  | -0.50  (0.620) |  | -0.51  (0.610) |
| Education (Base: Less than junior high school) |  |  |  |  |  |
| Junior high school |  |  | –0.27  (0.786) |  | 1.40  (0.160) |
| Senior high school and above |  |  | –0.29  (0.770) |  | –0.94  (0.349) |
| Self-assessed health status (Base: Poor) |  |  |  |  |  |
| Relatively poor |  |  |  | 2.09**  (0.037) | 1.92*  (0.055) |
| Fair |  |  |  | 7.22***  (<0.001) | 7.04***  (<0.001) |
| Relatively good |  |  |  | 10.07***  (<0.001) | 9.80***  (<0.001) |
| Good |  |  |  | 9.12***  (<0.001) | 9.09***  (<0.001) |
| Smoking status (Base: Smoking) |  |  |  |  |  |
| Have quit smoking |  |  |  | 4.29***  (<0.001) | 4.24***  (<0.001) |
| Not smoking |  |  |  | 3.23***  (0.001) | 1.03  (0.302) |
| Observations | 12116 | 12116 | 12116 | 12116 | 12116 |
| R-squared | 0.0001 | 0.0016 | 0.0005 | 0.0187 | 0.0195 |

Note: The dependent variable is a binary variable indicating whether the respondent has Malignant tumor (=1 if has Malignant tumor and 0 otherwise). OLS: ordinary least squares. Model 1 includes adequate health literacy. Model 2: gender, age group, and household income in addition to the variable in Model 1. Model 3: occupation and education in addition to the variable in Model 1. Model 4: self-assessed health status and smoking status in addition to the variable in Model 1. Model 5: variables in Models 1–4. Estimates of the constants have not been reported. ***p < 0.01, ** p < 0.05, * p < 0.1. Standard errors are shown in parentheses.
